# The Postgraduate Educational Environment at a United Kingdom Dental School: A Quantitative Study Using a Modified Dundee Ready Educational Environment Measure Questionnaire

**DOI:** 10.1111/eje.13091

**Published:** 2025-03-24

**Authors:** Jennifer A. Haworth, Sam D. Leary, Patricia Neville, Julie C. Williams, Jonathon Schofield, David Dymock, Peter Fowler

**Affiliations:** ^1^ Bristol Dental School Bristol UK

**Keywords:** dental, DREEM, postgraduate

## Abstract

**Introduction:**

Quantitative research on postgraduate (PG) dental students' perceptions of their educational environment is lacking. The aim of this research was to increase our understanding of taught PG dental students' educational environment.

**Methods:**

A modified Dundee Ready Educational Environment Measure (DREEM) questionnaire was distributed to PG students at the University of Bristol Dental School. DREEM scores in relation to five domains (learning, teaching, academic, atmosphere and social), as well as total DREEM scores, were derived. In addition, demographic and global questions relating to blended learning and teaching support were captured. DREEM domain and total scores were summarised using medians, interquartile ranges and full ranges. Demographic data and answers to additional global questions were categorised as frequencies and percentages.

**Results:**

Thirty‐four participants (50% response rate) from four different taught PG programmes responded. Total DREEM scores suggest most PG students' perceived educational environment to be ‘excellent’ (55.9%) or ‘more positive than negative’ (41.2%). The highest scoring domain was teaching, while the lowest scoring was social. Global questions indicated good teaching support and acceptance of blending learning, although the reduced face to face peer/teacher contact did not meet the needs of some (20.5%) participants.

**Conclusions:**

Although the DREEM measure has been used widely in dental training, its use has been limited in PG dental settings. The teaching and learning environment at the University of Bristol was rated highly. The lower social scoring highlights potential deficiencies within the educational environment of these PG students.

## 1 Introduction

Undertaking postgraduate studies, in any discipline, is widely recognised as a pathway to personal and professional career development. In healthcare, there is an implicit assumption that knowledge and skills development is a lifelong and continuous process. Writing in 1900, Sir William Ostler noted, ‘If the license to practice meant the completion of his(her) education how sad it would be for the practitioner, how distressing to his(her) patient!’ [1] While specialisation is not a requisite for dental practitioners, demand for postgraduate training (PGT) has increased in the US (American Dental Association, 2021 cited in Bates et al. [2]) and internationally [3, 4, 5]. In 2017, four in 10 general dental practitioners had a postgraduate qualification in the UK [6]. There are studies to support the view that postgraduate studies improve clinical confidence among general dental practitioners [7].

The concept of an educational environment is complex but can broadly embrace the how, why and what students learn [8], as well as the educational climate and academic culture [2]. The educational environment encompasses many different settings and includes various social, cultural, intellectual and material contexts. Students also receive education in an environment with a set of values [9]. This educational environment can be described as the atmosphere perceived by the students and teachers and is considered central to health professional education [10]. This atmosphere plays an important role in the health professional's engagement and ability to learn and is related to their achievements, satisfaction and success [11].

There is evidence that perceived positive educational environments lead to improved learning experiences and academic outcomes, while perceived negative educational environments lead to poorer academic outcomes [12]. Other evidence has linked the educational environment with student success and wellbeing [9], while perceived stress within the clinical educational environment for dental postgraduates has been linked with burnout [13]. Furthermore, it has been reported that the clinical postgraduate educational environment impacts both learning experiences and future clinical practice [14]. Although investigating the students' perceptions of their educational environment is considered important, the literature reporting on dental postgraduate students' perceptions is limited [15].

Different methodologies have been undertaken to investigate dental postgraduate students' perceptions of their educational environment involving qualitative, quantitative and mixed methods [16, 17, 18, 19]. Most dental schools actively seek regular formal and informal feedback from their postgraduate students as part of quality assurance and this informs adjustments to pedagogical activities. Problems with engagement and difficulties gaining meaningful representative feedback can prevent formal assessments and benchmarking of the postgraduate educational environment. Recent recommendations from the Advance Higher Education Academy include gaining greater insights into the educational environment beyond student satisfaction‐based feedback by accessing multiple data sources [20]. This is particularly important when disruptions are imposed, such as those experienced during the recent Covid pandemic, moving to new facilities and national dental postgraduate curriculum changes [21, 22, 23].

Although quantitative postgraduate educational environment measures have been developed such as the Postgraduate Hospital Educational Environment Measure (PHEEM), the Anaesthetic Theatre Educational Environment Measure (ATEEM) and the Surgical Theatre Educational Environment Measure (STEEM), these are specific to postgraduate medical settings and there is an absence of specific postgraduate measures for dentistry [10]. The Dundee Ready Educational Environment Measure (DREEM) is a generic quantitative tool developed for the assessment of the educational environment from the health science student's perspective [24]. Although DREEM has been widely adapted and used extensively in many different dental school locations and settings [25, 26, 27, 28, 29, 30, 31], its use in postgraduate dental settings has to date been limited [17, 32].

DREEM is a 50‐question closed questionnaire of the students' perception of their educational environment overall, and for five domains: learning, teaching, academic, atmosphere and social [24]. Although DREEM has been used to benchmark and monitor improvements undertaken in the individual domains, as well as identify course strengths and weaknesses [33], careful consideration of data analysis and reporting is required to allow meaningful study comparisons [34]. The validity and reliability of DREEM have been confirmed in postgraduate settings [35, 36].

Bristol Dental School is ranked fourth out of 16 in the UK (Complete University Guide Subject Rankings 2024) and currently hosts four PGT programmes for 68 students: MSc (Masters of Science) Implantology, DDS (Doctorate of Dental Surgery) Orthodontics, MSc Oral Medicine, PG (postgraduate) Certificate in Clinical Oral Surgery. Two of the programmes are full time (DDS Orthodontics and MSc Oral Medicine) with the DDS satisfying the research component of the orthodontic specialty training route, leading to specialist registration with the General Dental Council (GDC), whilst the MSc Oral Medicine, along with two part‐time programmes (MSc Implantology and PG Certificate of Oral Surgery) provides a qualification of special interest. Two additional PGT training programmes (MSc Periodontology and PG Diploma Orthodontic Therapy) have only recently commenced at Bristol Dental School.

During the Covid19 pandemic, there were significant challenges and changes to the postgraduate educational environment at Bristol Dental School, including a shift to blended teaching of academic material, a more restrictive research subject participation, reduced clinical load, increased personal protective equipment requirements including mask wearing during teaching and restricted aerosol‐producing clinical activities [22, 37]. Although many of these restrictions have been removed, the shift to blended learning has been integrated into academic teaching [38]. During this period, changes to the dental facilities were undertaken with the establishment of an additional second new geographical location for the dental school. Furthermore, potentially significant future changes to the specialist national training curriculum for postgraduate students were flagged by the GDC [39].

Current work undertaken to evaluate the postgraduate learning environment at the Dental School as part of the institution's good educational practice is limited to postgraduate programme‐specific mid‐term and annual student feedback surveys, which in turn feed into the dental school's Educational Action Plans. However, to investigate and provide greater in‐depth understanding of the educational environment as well as to establish a benchmark to allow monitoring of the impacts of future changes imposed, a quantitative assessment was undertaken using a modified online DREEM questionnaire. These findings will also inform future educational strategy for postgraduate students at Bristol Dental School. The aims were to investigate and describe the educational environment of a cohort of postgraduate students' perceptions of their training at Bristol Dental School and establish baseline data for future longitudinal investigations.

## 2 Methods and Materials

### 2.1 Participants and Setting

This was a cross‐sectional study at Bristol Dental School, University of Bristol, involving the distribution of a modified online DREEM questionnaire. Ethical approval was granted by the Faculty of Health Science Research Ethics Committee at the University of Bristol (reference number 12806).

The questionnaire was distributed via email by the training programme directors of the following postgraduate courses: MSc Implantology, DDS Orthodontics, MSc Oral Medicine, PG Certificate in Clinical Oral Surgery. The email invitation contained a participant information sheet and a description of the study, with a link to the online questionnaire. Having followed the online link, it was required that participants confirm that they had read a consent statement prior to continuing to the rest of the questionnaire. Data were collected between March and April 2023. The questionnaire was hosted on the “Online Surveys” platform. At the end of the survey, there was an option to provide a contact email address to be entered into a prize draw for two shopping vouchers of £100 each. Any email addresses provided were not used to link to survey data.

### 2.2 Outcome Measures

A copy of the questionnaire is available for reference via the following link postgraduate modified DREEM questionnaire. The questionnaire was divided into two sections: the first part including demographic and circumstantial questions (q1–15) and the second part containing DREEM questions (q16) as well as an additional eight final questions relating to the postgraduate dental environment setting. The questionnaire was piloted by the authors.

Demographic and circumstantial information was captured by multiple choice answers (q1–15), including information about the student's sex, ethnicity and nationality. Postcode data were collected for inference of Index of Multiple Deprivation data. Information was also collected regarding marital status, family dependents, accommodation, employment status, PGT programme, year of study within the course and prior clinical and research experience.

The second part of the questionnaire (q16) was based on the DREEM questionnaire, but modified by the authors of this paper where appropriate to make the questionnaire relevant for postgraduate dental students. The original DREEM question ‘Much of what I have to learn seems relevant to a career in medicine’ was changed to ‘Much of what I have to learn seems relevant to my future career’. ‘I am confident about passing this year’ was changed to ‘I am confident about passing’. ‘The enjoyment outweighs the stress of studying medicine’ was changed to ‘The enjoyment outweighs the stress of studying’. ‘The atmosphere is relaxed during the ward teaching’ was changed to ‘The atmosphere is relaxed during the clinical teaching’. ‘This school is well timetabled’ was changed to ‘This course is well timetabled’. ‘Cheating is a problem in this school’ was changed to ‘Cheating is a problem on this course’. ‘I have good friends in this school’ was changed to ‘I have good friends in this course’.

The final additional eight questions captured information about the teaching and support required to develop research, clinical and academic skills, as well as questions about blended learning, communication about course changes and support for individual health and wellbeing; these are referred to as global questions. These global questions were added and DREEM modifications made to reflect the postgraduate dental environment setting and to identify strengths and weaknesses in learning and wellbeing support. The questions were scored using a Likert scale of five options: 4 = strongly agree, 3 = agree, 2 = uncertain, 1 = disagree and 0 = strongly disagree. The maximum total DREEM score was 200 and sub scores were calculated for each of the five domains, with these domains being learning, teaching, academic, atmosphere and social. These domain questions were derived from the DREEM, published by Roff et al. [24] and relate to q16 of our questionnaire, except the final eight global questions. The five domain scores and total score were converted to percentages. In addition, a categorical variable was created based on the raw total DREEM score, with categories defined as very poor (0–50), problematic (51–100), more positive than negative (101–150) and excellent (151–200) [31]. Nine of the questions within the DREEM questionnaire were reversed for validation purposes and the scores adjusted accordingly.

### 2.3 Statistical Analysis

All analyses were undertaken in Stata version 17 (StataCorp). First, the characteristics of those who were included in this study were summarised as frequencies and percentages. The percentage of males in the study was compared to all students enrolled on the four postgraduate programmes. Index of Multiple Deprivation data from all countries of the British Isles were combined and presented as two categorical groups, with the highest 50% of scores placed into the ‘less deprived’ group and the lowest 50% of scores placed in the ‘more deprived’ group. The five domain and total scores derived from the DREEM questionnaire had skewed distributions, so were summarised using medians, interquartile ranges (IQRs) and full ranges. The categorised total score was summarised using frequencies and percentages, as were the final eight global questions (collapsed into strongly disagree/disagree, uncertain or agree/strongly agree).

## 3 Results

The following numbers of students completed the study: 6/8 on the DDS(Orthodontics), 2/2 on the MSc(Oral Medicine), 20/46 on the MSc(Implantology) and 6/12 on the PG Cert(Oral Surgery) programmes, so the overall response rate was 50%. The percentage of males in the study was lower compared to the whole student cohort (52.9% vs. 60.0%).

The characteristics of the 34 PG students who were included in the analysis are shown in Table 1. Just over one fifth (20.6%) were international students. Half of the students (50.0%) fell into the ‘less deprived’ category. Over two thirds (70.6%) were married or had a partner, and 41.2% had dependents including children, parents and/or siblings. Just under two thirds (64.7%) lived in independent or rented accommodation, as opposed to with family. Just under two thirds (64.7%) were self‐employed, whilst the rest were either employed (26.5%) or full‐time students (8.8%). Just over three quarters (76.5%) were on part‐time programmes, and a similar percentage (79.4%) were in the first year of their programme. Prior to starting their PG programmes, 58.8% had more than 5 years' clinical experience, 23.5% had a Master's degree or Doctorate and 47.1% had written a formal dissertation and/or published peer‐reviewed articles.

**TABLE 1:** Study participant characteristics (*N* = 34).

Characteristics	Subgroups	*N* (%)
Sex	Male	18 (52.9)
	Female	15 (44.1)
	Prefer not to say	1 (2.9)
Ethnicity	White	18 (52.9)
	Other	16 (47.1)
Nationality	International	7 (20.6)
	UK	27 (79.4)
Deprivation score^a^	More deprived	16 (47.1)
	Less deprived	17 (50.0)
	Unknown	1 (2.9)
Marital status	Single	10 (29.4)
	Married/partnered	24 (70.6)
Family dependents	Yes	14 (41.2)
	No	20 (58.8)
Accommodation	At family home	12 (35.3)
	Independent/rented	22 (64.7)
Work situation	Self‐employed	22 (64.7)
	Employed	9 (26.5)
	Neither	3 (8.8)
PGT programme	Full time	
	DDS (Orthodontics)	6 (17.7)
	MSc (Oral Medicine)	2 (5.9)
	Part time	
	MSc (Implantology)	20 (58.8)
	PG Cert (Oral Surgery)	6 (17.7)
Year of study	1st year	27 (79.4)
	2nd year	1 (2.9)
	3rd year	6 (17.7)
Pre PGT programme experiences	Clinical experience	
	1–5 years	14 (41.2)
	> 5 years	20 (58.8)
	Academic experience	
	Batchelor	26 (76.5)
	Masters/Doctorate	8 (23.5)
	Research experience	
	None	18 (52.9)
	Publications/dissertation	16 (47.1)

*Note:* Postgraduate taught (PGT); Doctor of dental surgery (DDS) in Orthodontics is a 3 year full‐time programme; Masters of Science (MSc) in Oral Medicine is a 1 year full‐time programme; MSc in Implantology is a 3 year part‐time programme; Postgraduate certificate (PG Cert) in Oral Surgery is a 1 year part‐time programme.

^a^ Deprivation score was derived from postcode data using the Index of Multiple Deprivation and presented as two categorical groups, with the highest 50% of scores placed into the ‘less deprived’ group and the lowest 50% of scores placed in the ‘more deprived’ group.

The five domain and total scores based on the DREEM questionnaire are summarised in Table 2a. The highest scoring domain was teaching (88.6%), followed by atmosphere (81.3%), academic (73.4%), learning (72.9%) and then social (67.9%). The lower score in the social domain looked to be mainly driven by a relatively high percentage of students feeling tired, as well as around a quarter of students being uncertain as to whether they felt their accommodation was pleasant and/or their social life was good and a third being uncertain about the support system for students who get stressed (Table 2b).

**TABLE 2:** (a) Students' perceptions of the learning environment in relation to each of the five DREEM domains (learning, teaching, academic, atmosphere and social) and total scores. (b) Students' perceptions of the learning environment in relation to the DREEM Domain 5 (Social).

(a)					
DREEM	Domain percentage score	Interquartile Range		Range %	
Domain	Median %	25th centile	75th centile	Minimum %	Maximum %
DREEM Domain 1 (Learning)	72.9	66.7	89.6	41.7	95.8
DREEM Domain 2 (Teaching)	88.6	77.3	93.2	54.5	100.0
DREEM Domain 3 (Academic)	73.4	62.5	78.1	40.6	100.0
DREEM Domain 4 (Atmosphere)	81.3	68.8	89.6	47.9	100.0
DREEM Domain 5 (Social)	67.9	53.6	78.6	32.1	96.4
Total	77.5	69.0	84.5	47.5	96.5

(b)			
Item	Disagree or strongly disagree %	Uncertain %	Agree or strongly agree %
Q16.44 I have good friends in this course	11.8	11.8	76.4
Q16.45 There is a good support system for students who get stressed	5.9	35.3	58.8
Q16.46 I am too tired to enjoy this course	41.2	14.7	44.1
Q16.47 I am rarely bored on this course	14.7	11.8	73.5
Q16.48 My accommodation is pleasant	5.4	26.8	67.8
Q16.49 My social life is good	5.9	23.5	70.6
Q16.50 I seldom feel lonely	14.7	20.6	64.7

The median (IQR) total score was 77.5 (69.0, 84.5)% (equating to a total DREEM score of 155 out of a maximum of 200); with 55.9% excellent, 41.2% more positive than negative and 2.9% plenty of problems.

The eight global questions are summarised in Table 3. At least two thirds of students agreed/strongly agreed with all the statements except that the reduced direct peer and staff contact had met their needs, for which 44.2% agreed. Less than 10% disagreed/strongly disagreed with all the statements except that 20.5% did not feel that the reduced direct peer and staff contact had met their needs, and 11.8% did not feel that any changes in the course or teaching had been communicated effectively.

**TABLE 3:** Global question responses related to blended learning, support for health and wellbeing, and for development of research, clinical and academic skills.

Global questions	Responses *n* (%)		
	Disagree or strongly disagree	Uncertain	Agree or strongly agree
Q16.51 Changes to blended learning have met my needs	2 (5.9)	11 (32.3)	21 (61.8)
Q16.52 The reduced direct peer and staff contact has met my needs	7 (20.5)	12 (35.3)	15 (44.2)
Q16.53 Any changes in the course or teaching have been communicated effectively	4 (11.8)	4 (11.8)	26 (76.4)
Q16.54 I receive adequate support and resources for blending learning	0 (0.0)	6 (17.7)	28 (82.4)
Q16.55 I receive adequate support for health and wellbeing	1 (2.9)	8 (23.5)	25 (73.5)
Q16.56 The teaching and support to develop advanced research skills has met my needs	2 (5.9)	10 (29.5)	23 (67.6)
Q16.57 The teaching and support to develop specialist clinical skills has met my needs	3 (8.8)	4 (11.8)	27 (79.4)
Q16.58 The teaching and support to develop specialist academic skills has met my needs	2 (5.9)	4 (11.8)	28 (82.3)

## 4 Discussion

This cross‐sectional survey measured perceptions of the learning environment of postgraduate dental students at Bristol Dental School. The response rate (50%) was comparable with the large DREEM study of dental undergraduate students based at Peninsula Dental School (56.4%), published in 2012 [25], although response rates for the DREEM questionnaire in the literature vary widely, from 18% [40] to 90% [31]. The DREEM tool has been used in both dental settings and in postgraduate settings and has proven utility for the dental education environment [26].

The demographics of the sample were slightly different from the postgraduate dental student population at Bristol Dental School, with a lower proportion of males responding to the survey compared to the overall population. This is largely a result of a proportionally lower survey uptake from the Implantology MSc students, with 78% of this cohort being male. Another limitation of this study is the small sample size, reflecting the small postgraduate dental cohort at Bristol Dental School.

The students were overwhelmingly positive about the overall learning environment, with the DREEM results indicating the vast majority perceived their learning environment to be either excellent or more positive than negative. This is important as student perceptions of their learning environment have been found to be significant predictors of wellbeing [41]. The overall positive results in this study are comparable to several publications employing DREEM at the undergraduate level in dentistry [25, 26, 27, 28]. The teaching domain received the highest scores and the social domain scored the lowest in our study. In an Australian dental student DREEM study, academic and social domains scored the lowest [31] and low scores for the DREEM question ‘social life is good’ were captured for dental students in five institutions in Pakistan [42]. Social domain also scored poorly in a DREEM survey of 1205 dental undergraduate students across three universities in Syria [43]. The UK‐based DREEM survey of over one hundred undergraduate dental students at the newly formed Peninsula Dental School [25] reported DREEM scores of higher than average when compared to international studies. The authors also reported that the scores for stress and social self‐perception domain were higher than traditionally found in other studies, proposing that the academic and pastoral support available to students helped in this domain, as well as the heterogeneous educational background of their graduate entry students [25].

There is no known published work examining DREEM scores of dental students in the UK at the postgraduate level. A Brazilian study of postgraduate dental students (in periodontology) employed DREEM to capture perceptions of the learning environment. They reported their mean total DREEM score to be 146.8 [44], lower than the median total DREEM score in this study of 155. The mean DREEM score in this study was 153.6, but this is a less appropriate summary than the median as the data are skewed. The authors quoted 55% of their students considered their social environment as ‘not a nice place’ [44]. The postgraduate dental learning environment in a teaching hospital in Nigeria was an environment perceived as having some problems [17] with a total mean DREEM score of 52.7% and a mean score of 47.4% for the social self‐perception domain, both scoring lower than the median scores of 77.5% and 67.9%, respectively, in our study. Caution is required when attempting to draw direct comparisons between studies employing the DREEM tool, as there are large disparities between countries in terms of education resourcing. In addition, reporting and statistical analysis varies between publications [34].

This study has highlighted the lowest scoring domain being the social domain. Of particular note was the 44.1% of the cohort either agreeing or strongly agreeing that they are too tired to enjoy their course. Although all participants in this study were undertaking clinical‐based studies, 91.2% were part‐time students who were either self‐employed or employed with other additional work commitments and 41.2% reported having home‐based dependants. Indicators of tiredness, loneliness, boredom and lack of social life may indicate signs of stress or lack of wellbeing. Stormon et al. reported that students who perceived the dentistry environment negatively had a 3.5 times increased odds of stress [41] and 40% of postgraduate dental students were reported to have ‘burnout’ in a Greek study, with students on clinical programmes showing increased levels of stress compared to non‐clinical or PhD programmes [13].

Another finding of this study, particularly relevant to social interactions, is that only 44.2% of participants agreed or strongly agreed with the statement that the reduced direct peer and staff contact had met their needs. Despite the many advantages that online teaching and learning can bring, including convenience and reduced need to travel, traditional face‐to‐face teaching still must be the cornerstone of dental education [45]. The advantages of peer‐to‐peer contact associated with teaching and learning in a physical location must not be underestimated.

Heeding the dental student voice or perspective is important, regarding students as collaborative partners in the educational process [46] and the findings of this study will be used at the programme and School level to inform change. The change in location of the Dental School at Bristol may have had a detrimental effect on the perceived sense of community for some postgraduate dental students, as many are now spread across different sites and work is ongoing to improve engagement at a postgraduate level in both taught and research programmes. It is planned that the results from this project will provide baseline data for subsequent comparisons in future years, following the introduction of interventions to establish an improved sense of community. The validity and reliability of students' evaluation of teaching practices have been questioned [15]; therefore, a further qualitative study, examining clinical, research and academic components of the postgraduate courses is currently being prepared for publication.

## 5 Conclusion

This study showed that postgraduate dental students at Bristol Dental School were positive about their educational environment. The domain that scored lowest was the social domain, with more than 40% too tired to enjoy their course and less than half of students agreeing that reduced direct peer and staff contact has met their needs. The findings of this study will be used to implement changes to the postgraduate learning environment at Bristol Dental School, particularly in relation to improving the social learning environment for students.

## Author Contributions

All the authors contributed to the development of the questionnaire‐based survey. P.F. and S.D.L. were responsible for data collection and analysis. P.F., S.D.L., J.A.H. and P.N. drafted the manuscript. All the authors read, revised and approved the final manuscript.

## Conflicts of Interest

The authors declare no conflicts of interest.

## Data Availability

The data that support the findings of this study are available on request from the corresponding author.
